# An International Expert Consensus Statement and Guidance Document on Implementation of Nutritional Supplementation in Patients on Dialysis

**DOI:** 10.1016/j.ekir.2026.106990

**Published:** 2026-07-29

**Authors:** Angela Yee-Moon Wang, Denis Fouque, Mary Rose Bisquera, Martin K. Kuhlmann, Lim Soo Kun, Kelly Lambert, Paola V. Miranda Alatriste, Kearkiat Praditpornsilpa, Alice Sabatino, Yew Shiong Shiong, Pablo Molina

**Affiliations:** 1 Department of Renal Medicine, Singapore General Hospital, Singapore; 2 Duke-National University of Singapore (NUS), Singapore; 3 Department of Nephrology-Dialysis, Hôpital Lyon Sud and University Claude Bernard Lyon 1, Lyon, France; 4 Department of Medicine, Section of Nephrology, Makati Medical Center, Manila, Philippines; 5 Department of Internal Medicine, Division of Nephrology, Vivantes Klinikum im Friedrichshain, Berlin, Germany; 6 Renal Division, Department of Medicine, University of Malaya, Kuala Lumpur, Malaysia; 7 School of Medical, Indigenous and Health Sciences, University of Wollongong, New South Wales, Australia; 8 Department of Nephrology and Mineral Metabolism, National Institute of Medical Sciences and Nutrition Salvador Zubirán, Mexico City, Mexico; 9 Division of Nephrology, Department of Medicine, Faculty of Medicine, Chulalongkorn University, Bangkok, Thailand; 10 Division of Renal Medicine, Department of Clinical Science Intervention and Technology, Karolinska Institutet, Stockholm, Sweden; 11 Department of Nephrology, Mahkota Medical Centre, Melaka, Malaysia; 12 Department of Nephrology, Hospital Universitari i Politècnic La Fe, Metabolism in Renal Disease (MET-REN) Research Group, Health Research Institute La Fe (IIS La Fe), Valencia, Spain; 13 Department of Medicine, Universitat de València, Valencia, Spain

**Keywords:** consensus meeting, dialysis, intradialytic parenteral nutrition, nutritional support, oral nutritional supplements, renal nutrition

## Abstract

**Introduction:**

Although some international guidelines suggest treatment of protein-energy wasting (PEW) in patients receiving dialysis with oral or parenteral nutritional supplementation, there is a current lack of practical guidance on their implementation. This document, developed by an international expert group, aims to bridge this gap by providing evidence- and consensus-based statements for clinicians on the practical implementation of clinical nutrition in patients on dialysis.

**Methods:**

A globally diverse group consisting of 8 nephrologists and 3 dietitians developed this guidance document on oral nutritional supplementation (ONS) and intradialytic parenteral nutrition (PN; IDPN) in patients receiving dialysis. The group started with 46 statements, which were drawn up before 2 parallel on-site meetings in Kuala Lumpur and Frankfurt/Main in November 2024. These statements were discussed there, and after another round of anonymous review, were put up for anonymous voting, reaching a final stage of consensus statements using the method outlined by the European Society for Clinical Nutrition and Metabolism for informed best clinical practice.

**Results:**

Altogether, the 20 consensus statements and 34 practice points provide concise guidance to implement and execute nutritional support in patients receiving maintenance dialysis across clinical settings and national health care systems. The rationales for each statement outline the current evidence, interpret it in light of expert experience, and incorporate information gained from the authors’ clinical practice.

**Conclusion:**

Providing clinical nutrition to patients on dialysis requires a structured approach. Screening and regular nutritional assessment of all patients are the basis of this approach. The oral nutritional support strategy needs to consider patient preferences and may require renal-specific formulas. For patients with PEW, IDPN is a reliable nutritional support option that may be used in selected patients to supplement the oral diet or used in combination with ONS to help meet their nutritional requirements.

Patients with chronic kidney disease (CKD) and those receiving dialysis are at high risk for PEW as a result of increased protein degradation and reduced protein and energy intake, secondary to inflammation, uremia, acidosis, and other hypercatabolic complications.^1^^,^^2^ Other factors such as reduced physical activity, impaired anabolic capability, comorbidities (such as diabetes mellitus and heart disease), and advanced age^3^^,^^4^ may further contribute to a hypercatabolic status in patients on dialysis, placing them at a heightened risk of PEW. The prevalence of PEW in patients on dialysis has been estimated to be 28% to 54% based on a meta-analysis including reports from 90 clinical studies worldwide.^5^ These findings indicate that early nutritional screening and assessment are crucial to identify patients at high-risk. The diagnosis of PEW should be followed by the early initiation of nutritional counseling and nutritional supplementation.

There is a global shortage of dietitians or renal dietitians, especially in resource-restricted countries, to provide renal nutritional care or renal nutritional counseling.^6^ Nephrologist training also does not include nutritional management for patients with kidney disease, and many nephrologists may consider that they are not responsible for delivering nutritional management. As recommended by the Kidney Disease Outcomes Quality Initiative (KDOQI) and the Kidney Disease: Improving Global Outcomes guidelines, patients with CKD who have signs of PEW should be referred to renal dietitians or dietitians specialized in renal nutrition for medical nutrition therapy.^7^^,^^8^ Furthermore, the European Society of Parenteral and Enteral Nutrition guidelines emphasized the importance of the early initiation of nutritional support in patients hospitalized with CKD to decrease the risk of PEW and improve their functional independence and self-care ability.^1^

## Methods

To better align current guideline recommendations with practical implementation across the globe, a global panel of nephrologists and dietitians was assembled and convened to develop a practical guidance document on the implementation of medical nutrition therapy in patients receiving dialysis in clinical practice. The guidance statements were drawn up based on both clinical evidence and expert consensus for those aspects that lack strong and high-quality clinical evidence. The guidance document aims to raise awareness of the prevalent nutritional complications in patients with CKD and on dialysis, promote the uptake of clinical practice guidelines on nutritional management, and improve the practical implementation and prescription of nutritional supplementation that are often needed in patients with PEW or at risk of PEW. The scope of this document covers ONS and IDPN. The statements in this guidance document refer to patients on long-term maintenance dialysis, including hemodialysis and peritoneal dialysis.

An international group of 8 nephrologists and 3 dietitians was assembled. Each expert was assigned a topic that covered specific aspects of ONS and IDPN. The experts developed a presentation, which they recorded and presented online. Two on-site meetings, one in Kuala Lumpur (Malaysia) and one in Frankfurt (Germany), connected by a Zoom platform, were run simultaneously in parallel for discussions on November 8, 2024, to develop the guidance document with expert consensus statements. Based on the online presentations, the meeting chairs drafted statements for group review and discussion. The statements were reviewed and discussed before the live meetings by the expert group at each location and then jointly during the Zoom session with the entire faculty. After the live meetings, the chairs (AY-MW, DF, and PM) refined the statements and sent them to the faculty for review and comments. The chairs then developed a final version of each statement, which was voted on in a subsequent anonymous online survey. For the voting, each statement was presented alongside the relevant scientific evidence and rationale. Consensus was rated according to the method adopted by the European Society for Clinical Nutrition and Metabolism guidelines. In essence, “strong consensus” was defined as agreement by > 90% of participants; “consensus” was defined as agreement by > 75%–90% of participants; “majority agreement” was defined as agreement by > 50%–75% of participants; and “no consensus” was defined as agreement by < 50%.^1^

During the live discussion of the consensus statements, it was agreed unanimously by the group to provide practice points in addition to the consensus statements to facilitate the practical implementation of nutritional supplementation by health professionals. Major topics were voted upon by the group to ensure their applicability and relevance for clinical implementation.

Altogether, 20 consensus statements and 34 practice points were drawn up by the group (Supplementary Table S1), and their rationales are presented below.

### Part 1: ONS

ONS plays an important role in the prevention and treatment of PEW in patients on dialysis. It works by preventing the depletion of circulating plasma amino acids and maintaining or increasing serum albumin levels.^9^ At the same time, the additional energy provided by ONS reduces the use of proteins as an energy fuel, thereby minimizing protein catabolism. There are data to suggest that intradialytic ONS reduces the risk of all-cause hospitalizations in patients on dialysis with hypoalbuminemia.^10^

However, there are several barriers that limit the implementation of ONS in clinical practice. First, nephrologists and renal nurses often have low awareness of changes in patient’s nutritional status,^11^ and most centers or hospitals do not routinely perform nutritional screening in patients to detect those who are at nutritional risk or with PEW. Globally, there is a lack of renal dietitians or dietitians trained specifically in renal nutrition, and knowledge gaps further hamper renal nutritional care.^6^ Furthermore, in many countries, ONS is not reimbursed in either inpatient or outpatient settings and must be paid for out of pocket, which places an additional financial burden on patients (Supplementary Table S2).^6^

Thus, it is important to tailor ONS prescriptions according to patients’ nutritional needs. ONS, as a standard, includes macro- and micronutrients and provides not only energy and protein but also vitamins, minerals, and electrolytes. It is also important to consider patient-specific factors, such as their acceptance of a specific ONS in terms of composition, flavor, and consistency, to ensure adherence to ONS. It is also important to provide regular monitoring of patients’ nutritional status for those who are receiving ONS.

Supplementary Table S1 provides the consensus statements on ONS for patients receiving dialysis, including several practice points. We provide the rationale for these statements as follows:

1. **Nutrition Screening for Patients on Dialysis**

1.1 **All patients receiving dialysis should undergo regular nutritional screening at least once every 3 months. Those at risk of PEW should undergo more frequent nutritional screening. (Strong consensus, 100% agreement)**

#### Practice Points

• Nutritional screening may be performed by any health care professional who has undergone training.
• There is no evidence of superiority of any specific nutritional screening tool in patients receiving dialysis. Commonly used tools include the Geriatric Nutritional Risk Index, Malnutrition Universal Screening Tool, Malnutrition Screening Tool, and the Renal Inpatient Nutrition Screening Tool. The choice of nutritional screening tool depends on local expertise and capabilities.

#### Rationale

Patients on dialysis are often multimorbid, and comorbidities or complications further increase their risk of developing PEW. For example, infections or cardiovascular disease may directly compromise nutritional status. Other conditions, such as fluid overload or uremia, may compromise appetite and are associated with PEW.^12^^,^^13^ Patients with advanced CKD may experience a reduction or loss of appetite (anorexia) and reduced dietary intake even before dialysis is initiated, putting them at risk for PEW.^14^^,^^15^ Therefore, regular ongoing screening for nutritional risk or PEW is essential for patients receiving dialysis. Although some clinical guidelines such as the KDOQI guidelines recommend regular nutritional risk screening and monitoring, they do not specify the recommended frequency of monitoring.

1.2 **Those at risk of PEW or found positive with nutritional risk during screening should be referred to a renal dietitian or a dietitian trained in renal nutrition for a detailed nutritional assessment. (Strong consensus, 100% agreement)**

#### Practice Points

• Several nutritional tools may be used for nutritional assessment. The International Society of Renal Nutrition and Metabolismcriteria for PEW,^16^ the Malnutrition Inflammation Score (MIS),^17^ and the Subjective Global Assessment (SGA)^18^ are the most commonly used tools for nutritional assessment. The Global Leadership Initiative on Malnutrition criteria^19^ are more frequently applied to other populations such as older patients. Regardless of the tools used for nutritional screening and nutritional assessment, health care professionals should receive training before they apply the tools.
• To adopt the diagnostic criteria of International Society of Renal Nutrition and Metabolism, PEW is diagnosed in patients with at least 1 criterion in 3 of the 4 categories, with at least 1 biochemical parameter included in the diagnosis of PEW (Table 1).^20^

**Table 1:** ISRNM criteria for the identification of protein-energy wasting (PEW)

Category	Parameter
Serum chemistry	• Serum albumin < 38 g/l • Serum prealbumin < 300 mg/100 ml • Serum cholesterol < 100 mg/100 ml
Body mass	• BMI < 23 kg/m^2^ • Unintentional weight loss: – at least 5% over 3 mos or – 10% over 6 mos
Muscle mass	• Muscle wasting: reduced muscle mass – at least 5% over 3 mos, or – 10% over 6 mos • Reduction of appendicular skeletal muscle index (DEXA) – < 5.45 kg/m^2^ in women and – < 7.25 kg/m^2^ in men • Reduced mid-arm muscle circumference area (over 10% reduction relative to the 50th percentile of the reference population) • Low creatinine appearance
Dietary intake	• Unintentional low dietary protein intake < 0.80 g/kg/d for at least 2 mos • Unintentional low dietary energy intake < 25 kcal/kg/d for at least 2 mos

BMI, body mass index; DEXA, dual-energy X-ray absorptiometry; ISRNM, International Society of Renal Nutrition and Metabolism.

**Figure 1:**
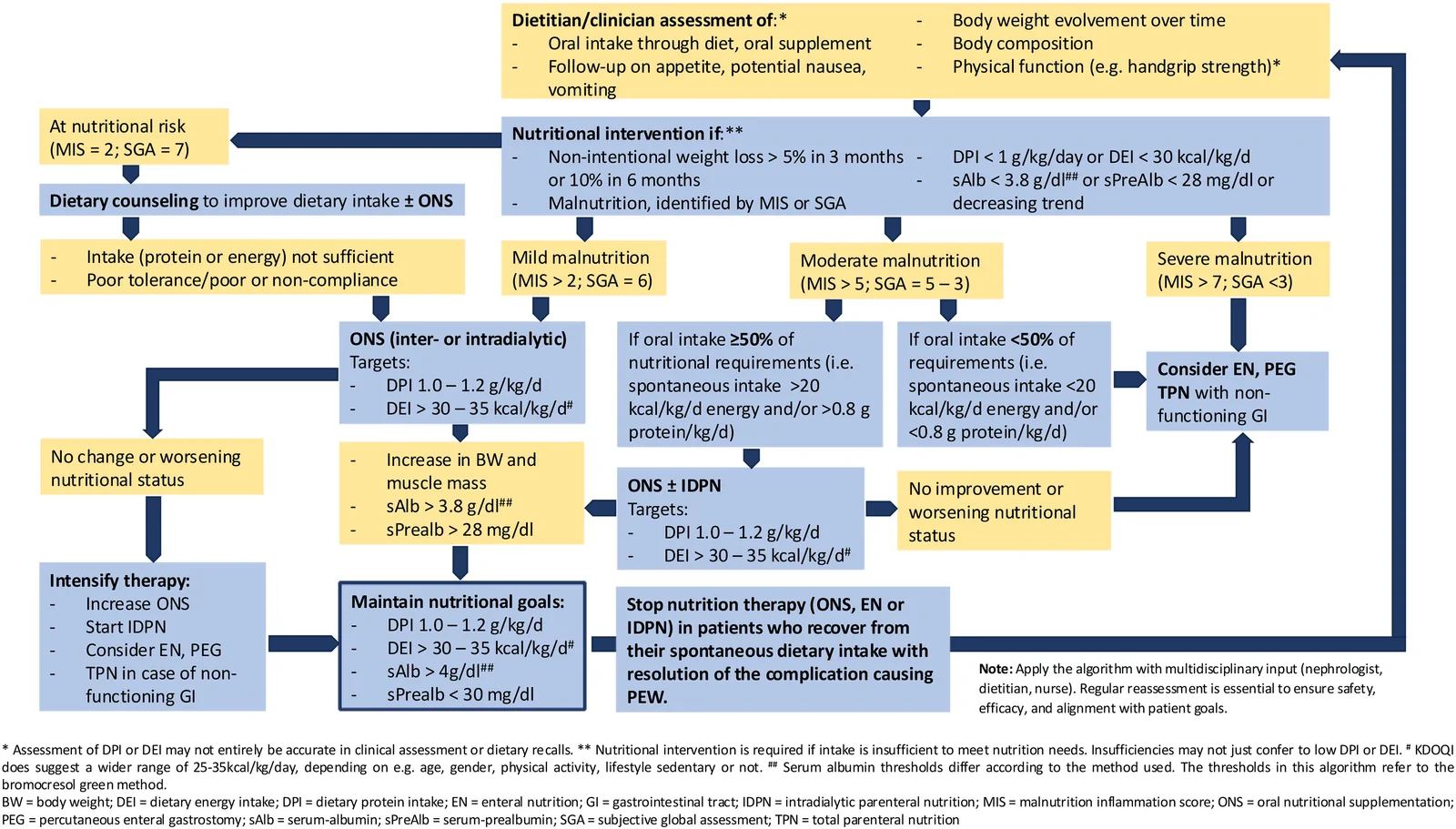
Implementation algorithm for nutritional support in patients receiving dialysis.
• Nutritional assessment should be performed by registered dietitians, renal dietitians, or their international equivalents, as suggested by international guidelines.^7^^,^^8^^,^^21^

A diagnosis of PEW requires at least 1 positive test result in at least 3 of the 4 categories, including at least 1 biochemical indicator.
• The use of the MIS or SGA, as presented in Figure 1, allows stratification of the severity of PEW and helps guide the intensity and type of nutritional intervention to be adopted according to severity. In this context, mild PEW may often be managed with nutritional counseling and ONS, whereas moderate-to-severe PEW may require escalation towards enteral nutrition (EN), IDPN, or total PN (TPN) depending on the clinical scenario, spontaneous oral intake, and the severity of hypercatabolic conditions.

#### Rationale

The 2 most widely validated (and clinically used) tools for nutritional assessment in patients receiving dialysis are the MIS and the SGA:

• MIS: The cutoff threshold of MIS >5 (on a 0–30 scale) is the most commonly used threshold to identify PEW in patients receiving dialysis, with higher scores indicating worse nutritional status.^22^^,^^23^ In a Spanish multicenter study of 2748 patients undergoing hemodialysis, 51.7% had PEW considering this threshold level.^23^ The MIS can be further stratified as follows: MIS ≤ 2 (normal), MIS > 2–5 (mild), MIS >5–7 (moderate), MIS > 7–10 (severe), and MIS >10 (extreme).^23^
• SGA: The SGA is graded on a 7-point scale, and a score of ≤5 is the standard threshold for identifying PEW in patients receiving dialysis, with lower scores indicating worse nutritional status.^24^^,^^25^ The 7-point scale is interpreted as follows: SGA scores of 6–7 (normal), SGA scores of 3–5 (mild-to-moderate), and SGA scores of 1–2 (severe).^8^ In a recent study of 105 patients receiving dialysis, 54.3% had PEW using an SGA score of ≤ 5 as the diagnostic threshold.^25^

Of note, the scores of these 2 assessment tools go in opposite directions—higher MIS scores indicate worse nutritional status, whereas lower SGA scores indicate worse nutritional status. Both are endorsed by the KDOQI 2020 guidelines, although they only show moderate agreement (κ = 0.43).^8^^,^^24^ The severity of PEW should be assessed and stratified, because it impacts subsequent nutritional intervention strategies, as outlined in Figure 1.

A global systematic review reported a high prevalence of PEW ranging between 28% and 54% among dialysis populations.^5^ Most of the studies included in the systematic review assessed nutritional status using the SGA or MIS. This indicates the need to raise awareness of the prevalent nutritional complications in patients on dialysis and to provide regular nutritional screening in this population to identify those at nutritional risk or with PEW for early intervention.^5^

2. **Nutritional Counseling**
2.1 **Patients at risk of or confirmed with PEW on nutritional assessment should receive nutritional counseling. (Strong consensus, 100% agreement)**

#### Rationale

The development of PEW is often multifactorial and involves both decreased nutritional intake and increased energy metabolism and protein degradation.^15^^,^^26^ In patients on dialysis complicated by PEW, preventing fat and muscle mass loss and preserving muscle mass are important goals of treatment.^27^

Frequent counseling by a dietitian may increase adherence to nutritional recommendations, helping to identify the causes of PEW.^3^ There are data from a retrospective cohort study in 75 patients receiving hemodialysis (mean MIS 7.8 ± 3.2), suggesting that nutritional counselling every 3 months (or more frequently if needed) may help to improve patients’ nutritional status, quality of life, and survival after 12 months.^21^ Another open, non-randomized, controlled study in 277 patients receiving maintenance hemodialysis showed that those who received nutritional counseling by renal dietitians twice per month for 6 months had a significantly better SGA score compared with baseline and with those who received counseling from other healthcare professionals.^28^

3. **Initiation of ONS**
3.1 **Consider initiating ONS early in patients with confirmed PEW who are unable to improve or increase their spontaneous dietary intake to meet nutritional requirements after nutritional counseling. ONS should supplement the nutritional needs of patients, which include energy, macronutrients, and micronutrients. (Strong Consensus, 100% Agreement)**

#### Practice Points

• Once a patient is diagnosed with PEW with reduced spontaneous oral intake that is insufficient to meet nutritional needs, ONS should be initiated.
• Patients who are prescribed ONS should be monitored for their tolerance of ONS, clinical progress, and nutritional status over time to tailor the formulation and prescribed amount accordingly.
• Generally, intake of ONS may provide around 250 to 300 kcal of energy per day.^29^
• It may be a challenge to administer more than 400 kcal/d orally.
• Not all ONS products are nutritionally complete; therefore, it is crucial to select an appropriate product based on the patient’s specific needs, clinical conditions, and any complications that may increase nutritional needs.^30^
• Concentrated ONS with reduced volume relative to their energy and protein content may result in a lower volume load for patients on dialysis and reduce the risk of volume overload.^31^
• Offering ONS products with similar nutrient content and volume but in a variety of flavors, consistencies, and formats, such as low-potassium or low-phosphorus drinks, energy or protein bars, and protein powders that can be mixed into puddings, juices, or shakes, may help reduce monotony and prevent taste fatigue, thereby enhancing adherence to ONS.
• Homemade ONS may be considered if commercial ONS is not available. It is important to provide close nutritional monitoring and education on best practices to safeguard adequate nutrient content and food safety.^32^
• Consider the patient’s preferences, gut tolerance, affordability, volume status, biochemical parameters, and comorbidities such as diabetes mellitus, heart failure, or volume overload when prescribing ONS for patients. The timing of ONS administration, whether intradialytic or interdialytic at home, should take into account the patient’s preference and the amount of energy and protein needed to meet their nutritional needs.

#### Rationale

ONS offers the most physiological way to supplement energy, protein, and other nutrients in patients on dialysis,^33^^,^^34^ including those who are elderly or frail.^35^ In order to ensure patient’s adherence to ONS, it is important to prescribe an ONS that patients prefer and tolerate.^3^ The KDOQI guidelines suggest that ONS prescriptions should take patient’s preferences into account.^8^ A randomized, controlled study of 86 malnourished patients on hemodialysis showed that providing ONS with nutritional counseling for 30 days resulted in higher dietary intake and reduced PEW, as reflected by a lower MIS, as opposed to providing nutritional counseling alone.^36^

A recent meta-analysis of 22 randomized, controlled studies showed that ONS consistently and significantly improved serum albumin levels,^37^ increased the normalized protein catabolic rate, and lowered MIS^34^ in patients on maintenance dialysis.^34^ Provision of ONS does not seem to increase the risk of gastrointestinal intolerance.^38^

4. **Renal-Specific ONS**
4.1 **Consider the use of high-protein, renal-specific ONS for patients receiving dialysis with PEW, as they provide more amino acids that are needed to maintain muscle mass and replenish dialysis-related losses, but with reduced volume. (Strong Consensus, 91% Agreement)**

#### Practice Points

• Patients on dialysis who are at risk of electrolyte imbalances or volume overload may benefit from a concentrated formulation of ONS that is low in specific electrolytes and reduced in volume.^8^
• ONS enriched with omega-3 polyunsaturated fatty acids (PUFAs), which may facilitate achieving intakes of 1.3 to 4 g/d of long-chain omega-3 PUFAs, could potentially support patients in meeting guideline recommendations to support blood lipid management. (Majority agreement, 73% agreement)

#### Rationale

A meta-analysis of 4 randomized controlled trials including 243 patients on hemodialysis who received amino acid supplementation did not identify a significant effect on muscle mass and muscle strength, but did show improved physical function as measured by the shuttle walk test, gait speed, and the timed up and go test.^39^ Three of the included studies in the meta-analysis lasted 6 months, suggesting that a 6-month duration may be needed for amino acid supplementation to be effective. Another randomized, controlled study reported improvements in serum albumin and prealbumin levels, higher body mass index, and a lower prevalence of PEW with the use of high-protein ONS, as compared with nutritional counseling.^40^ In general, high-protein ONS may provide around 15–30 g of protein per serving.

The KDOQI guidelines suggested a daily dosage of 1.3 to 4 g of long-chain omega-3 PUFAs may be prescribed to lower triglyceride and low density lipoprotein cholesterol levels and raise high density lipoprotein levels.^8^ Recent randomized-controlled nutritional studies suggested potential positive effects of oral omega-3 PUFAs on inflammation,^41^, ^42^, ^43^, ^44^ cardiovascular disease,^45^ pruritus,^46^ and nutritional status.^42^ These studies mostly used fish oil capsules containing 2 to 2.4 g/d eicosapentaenoic acid and docosahexaenoic acid, with a duration ranging from 8 weeks to 6 months. The recent randomized, placebo-controlled, double-blind Protection against Incidences of Serious Cardiovascular Events Study (PISCES) evaluated daily fish-oil supplementation (4 x 1.6 g eicosapentaenoic acid and 0.8 g docosahexaenoic acid per day) compared with corn-oil placebo in 1228 participants receiving hemodialysis for up to 3.5 years.^47^ It reported an approximately 40% lower rate of serious cardiovascular events (hazard ratio: 0.57; 95% confidence interval: 0.47–0.70; *P* < 0.001) in the fish oil group. In this study, 4.8% of patients in the fish oil group and 7.6% in the control group experienced serious bleeding events.

5. **Timing of ONS**
5.1 **ONS is preferably given after meals, during dialysis sessions, and/or late in the evening to avoid early satiety and to maximize nutrient absorption. (Strong Consensus, 100% Agreement)**

#### Practice Points

• It is preferable to administer ONS 1 hour after meals.^8^
• ONS may be considered a meal replacement to prevent PEW only in selected patients who are unable to meet their nutritional needs through conventional meals, including but not limited to those with swallowing difficulties. (Majority agreement, 73% agreement)

#### Rationale

It is recommended that ONS not be used to replace usual intake and instead are given away from meal times. Administering ONS during dialysis helps mitigate the known catabolic effects of the dialysis procedure, improves patient adherence to ONS, and minimizes the risk of patients having ONS in place of a main meal. Intradialytic oral or parenteral nutritional supplementation may compensate for the loss of amino acids and proteins during dialysis sessions^35^ and has been shown to improve MIS.^48^ A randomized, controlled, multicenter study in 100 patients receiving hemodialysis provided ONS (22 g protein powder) approximately 1 hour before the start of dialysis, compared with no supplementation. ONS significantly increased serum albumin levels and improved the Kidney Disease Quality of Life (KDOQI)-36 questionnaire scores across 3 subscales after 3 months of treatment.^49^

Although ONS can serve as a meal replacement, this option should only be reserved for very specific situations, such as those with swallowing difficulties, oral or buccal problems, intolerance to solid foods because of nausea, or extreme fatigue that prevents patients from managing a full meal. If possible, it is preferable to encourage patients to have their usual meal and use ONS as a nutritional supplement, because skipping their usual meals may increase the risk of PEW. One example of a useful strategy is to use 60 ml or 80 ml of the ONS with medications instead of water, 3 or 4 times daily.

For older patients, treatment goals may shift from prolonging survival to maximizing quality of life.^7^

6. **Monitoring of Tolerance**
6.1 **Tolerance and compliance with ONS should be closely monitored at its initiation. (Strong Consensus, 100% Agreement)**

#### Practice Point

• Patients should be monitored and reassured as needed, if temporary side effects occur. They should be able to receive advice regarding issues such as difficulty tolerating the taste or texture of ONS.

#### Rationale

In patients with PEW on hemodialysis, regular follow-up visits with a dietitian and regular monitoring of nutritional status may improve patient adherence and be useful to assess the effectiveness of nutritional supplementation.^3^ Additionally, it is important to avoid unnecessary fasting periods or diet restrictions, for example, during acute illness or hospital checkups.^3^

6.2 **In patients with poor gut tolerance or compliance issues with ONS, adjustment of the ONS formula, composition, and volume may be considered. (Strong Consensus, 100% Agreement)**

#### Rationale

The flavor, composition, and consistency of ONS may influence patient adherence to ONS and gut tolerability. A recent randomized, controlled study in patients receiving hemodialysis with PEW reported that pretesting available ONS products with the patients before initiation of the treatment may improve adherence to ONS supplementation.^50^

7. **Fiber Provision**
7.1 Patients **with PEW should receive dietary fiber either through diet, ONS or an additional fiber supplement, in accordance with guideline recommendations to promote overall gut health. (Strong Consensus, 100% Agreement)**

#### Practice Points

• The amount of fiber in the diet should be checked and supplemented if necessary. When initiating fiber-containing ONS, there is an increased likelihood of flatulence or gastrointestinal discomfort. Therefore, supplementation should begin gradually, starting with one serving per day.
• Validated methods to estimate patient’s fiber intake by dietitians include a 24-hour dietary recall and a 3 or 7-day food recall or diary.
• Total fiber intake should target the same amount as that recommended for the general population (25 to 30 g/per day, depending on the recommendation).

#### Rationale

Fiber is a critical component of the diet for gut health, especially as a source of microbial short-chain fatty acids.^51^ However, traditional advice to minimize nutrients such as potassium and phosphate has led to the elimination of many nutritious foods from the diet. This has now changed because of an improved understanding of nutrient bioavailability.

Chronic constipation is associated with a higher incidence of hyperkalemic episodes in patients transitioning to dialysis. ONS provides a reliable source of soluble fiber intake, and fiber may prevent constipation.^52^ Although evidence is limited, a meta-analysis of prospective cohort studies found a consistent inverse correlation between fiber intake and all-cause and cardiovascular mortality in patients with CKD, including those on hemodialysis or peritoneal dialysis.^53^ In fact, a prospective cohort study in patients receiving peritoneal dialysis using a 7-day food frequency questionnaire reported that every additional 1 g of fiber intake was associated with an 11% lower risk of major cardiovascular events.^54^

Recent controlled studies with patients on hemodialysis supplied 10 to 15 g of fiber per day, but it is not clear whether the studies accounted for other sources of fiber. In some of these studies, fiber supplements providing 9 to 50 g of fiber per day have been shown to significantly reduce uremic toxins, although the effect may be less pronounced in patients receiving dialysis (including peritoneal dialysis) than in patients with CKD not on dialysis. A meta-analysis of 21 randomized, controlled studies analyzing the effect of fiber supplementation on uremic toxins and inflammation did not find dose-dependent differences in a subgroup analysis comparing studies with fiber supplementation of up to 15 g/day and more than 15 g/day.^55^

8. **Enteral Nutrition**
8.1 **Consider the initiation of enteral nutrition in patients receiving dialysis who are severely malnourished and unable to reach > 20 kcal/kg/d energy intake or > 50% of estimated energy needs through spontaneous intake plus ONS. (Strong Consensus, 91% Agreement)**

#### Rationale

The KDOQI guidelines suggest that enteral tube feeding should be considered for patients with chronically inadequate nutritional intake and/or PEW whose protein and energy needs cannot be met by dietary intake and ONS.^8^ The consensus statement on EN by the American Society of Parenteral and Enteral Nutrition recommends the same approach in patients with CKD.^56^ The European Society for Clinical Nutrition and Metabolism guidelines for hospitalized patients with CKD recommend early nutritional support in patients with multiple chronic conditions and early enteral nutrition, if oral intake is not possible.^1^

The goal of combined oral and enteral nutritional supplementation is to achieve the recommended dietary energy intake of 25 to 35 kcal/kg ideal body weight per day for patients on dialysis with PEW, as confirmed by nutritional assessment.^8^

9. **Monitoring of Nutritional** **Support**
9.1 **Nutritional status should be monitored at least monthly after initiation of any form of nutritional** **support (ONS, EN, PN, IDPN, or TPN). (Strong consensus, 100% agreement)**

#### Practice Point

• After starting nutritional support, at least monthly monitoring is required. Nutritional monitoring can be integrated with tolerance checks. In patients receiving home dialysis (including PD and home HD), nutritional monitoring every 2 to 3 months may also be reasonable.

#### Rationale

The KDOQI guidelines recommend monitoring of nutritional status using body weight and body mass index at least monthly for all patients on dialysis (opinion).^8^ Changes in dry weight or BMI derived from dry weight after dialysis should be monitored. The reason to use dry weight is that total body weight is confounded by volume status. If patients are volume overloaded, this may mask weight loss. The nutritional assessment could be expanded to include other parameters such as appetite, dietary intake, body fat mass, muscle mass, and functional status to provide a more comprehensive picture of the patient’s overall nutritional status.

10. **Discontinuation of nutritional** **support**
10.1 **Duration of ONS should be tailored according to the causes of pew, clinical targets, and actual improvement. ONS is generally prescribed for a 3 to 6-month period as required. (Strong Consensus, 91% Agreement)**

#### Rationale

The KDOQI guidelines suggested a trial of ONS for 3 to 6 months in patients with PEW.^8^ The suggestion is based on the timeline needed to improve nutritional status with ONS in clinical studies. In a prospective, randomized, controlled study of patients undergoing hemodialysis or peritoneal dialysis who were at risk of or suffered from PEW, supplementation with renal-specific ONS under a dietitian supervision improved nutritional status after 3 months.^57^ Two meta-analyses of 22 randomized, controlled trials in patients on dialysis, with 10 studies included in both analyses, reported a significant increase in serum albumin after ONS treatment for studies lasting up to 6 months.^37^^,^^38^ No further improvement in nutritional status was observed when ONS was administered for over 6 months.

10.2 **Nutritional** **support (either ONS, EN, PN, IDPN, or TPN) may be discontinued when patients show an overall improvement in appetite, spontaneous dietary intake, weight gain, and improvement or resolution of the clinical condition causing PEW. (Strong consensus, 100% agreement)**

#### Practice Point

• The efficacy of ONS may be monitored using the parameters outlined in Table 2. Assessment of body composition is not mandatory but may assist in the follow-up of nutritional status and changes in body composition after nutritional supplementation.

**Table 2:** Parameters that can be used to assess and monitor nutritional status during ONS administration

Category	Parameter
Weight	• An increasing trend over the last 3 mos.
SGA/MIS	• Improvement in scores over the last 3 mos
3-d food diary/recall	• Spontaneous energy intake ≥ 25–30 kcal/kg BW/d • Spontaneous protein intake ≥ 1.0 g/kg BW/d
Predialysis serum albumin	• An increasing trend over the last 3 mos.

BW, body weight; MIS, Malnutrition-Inflammation Score; ONS, oral nutritional supplements; SGA, Subjective Global Assessment.

These parameters should be met with regular diet before stopping ONS.

#### Rationale

There are no recent studies evaluating weaning strategies for ONS. However, any nutritional support or therapy should have clearly defined goals. Generally, ONS may be stopped or tapered off if the acute complication causing PEW has resolved and patients are regaining their appetite, dietary intake, and body weight, and recovering their usual nutritional status. It is important to ensure that a patient’s nutritional needs are met by oral intake with or without ONS before an invasive form of nutritional supplementation, such as tube feeding, IDPN, or TPN is discontinued.

### Part 2: IDPN

IDPN is a type of PN provided during hemodialysis sessions. A notable benefit of IDPN is that it does not require additional venous access, because it can be infused through the venous chamber of the dialysis machine.^8^

Unlike TPN, IDPN is supplemental in nature and cannot provide total nutritional support.^58^ It is not a last-resort intervention for severely malnourished patients requiring full nutritional support, nor should it replace enteral nutrition in those with moderate to severe PEW. Although the nutrient content provided with IDPN may be comparable with that of ONS, IDPN may deliver higher amounts of energy and protein and is only given during hemodialysis sessions. It may avoid adherence issues related to intake of ONS. It is provided as a nutritional supplement to patient’s dietary intake, with or without ONS.

The most recent systematic review published in 2019^59^ examined studies using IDPN. The review included studies published between 2008 and 2018. There were 5 randomized controlled trials, 6 cohort studies, and 1 uncontrolled study. Although IDPN treatment regimens differed across studies, all but 3 studies were rated as having fair methodological quality. The majority of patients had received hemodialysis for at least 6 months, and the control groups mostly received “usual recommended diet”, without a detailed description. Patient’s nutritional status was evaluated by serum albumin along with at least 1 additional nutritional indicator, such as body weight loss, body mass index, or a nutritional score. Mean baseline serum albumin levels ranged from 3.02 to 3.8 g/dl.^60^ When compared with nutritional counseling, IDPN treatment improved some nutritional parameters, including serum albumin, serum prealbumin, SGA and MIS scores, body weight, and mid-arm circumference, but the heterogeneity of end points precluded a definitive conclusion. More recently, published randomized, controlled studies have demonstrated improvements in serum albumin,^61^^,^^62^ prealbumin,^63^ hemoglobin,^62^ muscle mass,^61^ body weight,^62^ and MIS after 3^61^^,^^62^ and 6 months of treatment with IDPN.^62^ Safety analysis demonstrated that adverse effects occurred during IDPN were generally similar to those in the control groups and were not clinically significant.^29^^,^^59^^,^^63^ The KDOQI guidelines suggested a trial of IDPN for patients with kidney failure receiving hemodialysis to improve PEW or maintain nutritional status if nutritional requirements cannot be met with spontaneous oral intake, ONS and/or enteral intake. However, the lack of randomized controlled trial evidence for IDPN, the absence of clear clinical guidelines or administration protocols, variability in IDPN formulations, and insufficient routine nutritional screening in dialysis centers, leading to underdiagnosis of PEW, pose significant barriers to its practical implementation.^64^^,^^65^ We provide the following consensus statements in relation to IDPN prescription for patients on hemodialysis (Supplementary Table S1).

11. **Initiation of** IDPN
11.1 **Initiate IDPN for patients with confirmed PEW whose spontaneous dietary intake is over half of the nutritional requirements but are unable to meet their total energy and protein requirement with ONS or enteral nutrition. (Strong consensus, 100% agreement)**

#### Practice Points

• Detailed criteria for the initiation of IDPN are listed in Supplementary Table S3.^66^ Commercially available formulations of IDPN containing all macronutrients (carbohydrate, fat, and protein) are preferred over individualized parenteral nutrition formulations prepared by the hospital pharmacy because the commercially available formulations generally meet the nutritional needs of most patients on hemodialysis. The standard formulations are immediately available and do not need to be specifically prepared. They require less preparation time, have a longer shelf life, and carry a lower risk of contamination. Because commercial formulations are adapted to patient’s electrolyte-free requirements and can provide high concentrations of nutrients, they help reduce the risk of electrolyte disturbances and the need for excessive ultrafiltration rates.^66^
• Suggested practical administration regimen: Initiate IDPN (triple-phase solution that contains fat, carbohydrate, and protein) at half the target volume during the first week and then gradually increase to full volume from weeks 2 to 4 according to patient’s clinical condition, nutritional status, volume status, glycemic control, electrolyte levels, and overall tolerability. (Strong consensus, 91% agreement). Table 3 shows the prescription volume, maximal infusion rates, and gradual step-up approach proposed by the experts.

**Table 3:** Infusion rates for IDPN solutions proposed in the literature

IDPN solution	Session duration	Week 1	Week 2	Source
650 ml	4 h	1.35 ml/kg/h (∼ 60–125 ml/h)	2.7 ml/kg/h (∼ 120–250 ml/h)	Molina *et al.*^66^
1000 ml	4 h	1.5 ml/kg/h (∼ 90–135 ml/h)	3.0 ml/kg/h (∼ 180–270 ml/h)	
Commercially available triple phase solution	4 h	125 ml/h	250 ml/h	Carrero *et al*^2^
500–1000 ml triple phase solutions	3–4 h	Up to 250 ml/h, depending on the formula and volume		Meade *et al.*^58^

IDPN, intradialytic parenteral nutrition.
• The inclusion of fish oil, a source of omega-3 PUFAs, in the IDPN formulation may be considered, as it could provide potential anti-inflammatory benefits and have a modest effect on triglyceride levels, although current evidence remains limited. (Consensus, 82% agreement)

#### Rationale

The role of IDPN is to bridge the nutritional gap between a patient’s nutritional needs and their actual dietary energy and protein intake, including ONS, in patients undergoing hemodialysis. Because it is provided during hemodialysis sessions, the amount is limited by the rate of administration, duration, and frequency of hemodialysis sessions.^8^ Owing to metabolic tolerance issues, IDPN should be delivered during a session lasting at least 3.5 to 4 hours.^66^

The target volume corresponds to the volume specified in the summary of product characteristics or patient information for a parenteral intradialytic nutrition formula suitable for patients. Table 3 shows the prescription volume, maximal infusion rates, and gradual step-up approach proposed by experts. The gradual step-up approach with IDPN prescription helps patients adapt from a state of PEW to one with reliable nutritional supply. Gradually increasing the IDPN volume is generally recommended in patients with PEW and poor feeding to prevent complications such as overfeeding syndrome, hyperglycemia, hypertriglyceridemia, gastrointestinal and hepatic issues, and refeeding-type shifts. This prescription approach also allows for the assessment of metabolic tolerance and better volume control.^2^^,^^66^^,^^67^

Commercial IDPN formulations may contain different lipid injectable emulsions, including traditional soybean oil-based emulsions, mixed-oil formulations (combining soybean oil, medium-chain triglycerides, olive oil, and fish oil), and, less commonly, pure fish oil-based emulsions. Soybean oil-based emulsions have historically been the standard; however, mixed-oil formulations are increasingly adopted because of their lower omega-6 fatty acid content and their potentially more favorable fatty acid profile. Although these theoretical advantages have led to the increasing use of mixed-oil formulations, there are currently no comparative studies in patients receiving IDPN demonstrating the superiority of one lipid emulsion over another. Similarly, evidence is lacking to support the preferential use of specific lipid emulsions according to individual patient characteristics (e.g., baseline hypertriglyceridemia).^68^, ^69^, ^70^, ^71^ Therefore, in the absence of comparative evidence in the IDPN setting, the choice of lipid emulsion is generally guided by local availability and institutional practice.^66^

Although direct comparative evidence on different lipid emulsions in IDPN is lacking, evidence from studies of omega-3 PUFA supplementation in patients receiving hemodialysis provides indirect support for the potential metabolic and anti-inflammatory effects of omega-3 PUFAs. A meta-analysis of 43 randomized controlled trials in patients on hemodialysis has shown that nutritional supplementation with omega-3 PUFAs was associated with a reduction in serum triglycerides, low density lipoprotein-cholesterol, and the inflammatory index levels.^72^ There is observational evidence showing a direct correlation between serum omega-3-PUFA levels and the risk of sudden cardiac death in incident patients undergoing hemodialysis.^73^ Additionally, a meta-analysis of 3 studies involving 514 patients on hemodialysis who received omega-3 PUFA supplements (1.7–4 g/d) indicates a trend toward a decreased incidence of cardiovascular events.^74^ These findings have been confirmed in the randomized, controlled, double-blind PISCES study, where a supplementation of 4 x 1 fish oil capsule per day reduced the rate of serious cardiovascular events significantly by approximately 40%.^47^

In a randomized, controlled study, IDPN containing omega-3 PUFAs from fish oil (with a mean delivery dose of 4.5 ± 0.6 g per infusion) was provided to patients on hemodialysis with PEW who had a spontaneous energy and protein intake of at least 20 kcal/kg/day and 0.8 g/kg/day, respectively. After 3 months of IDPN, serum albumin levels significantly increased compared with a control group receiving nutritional counseling only. Spontaneous oral intake also increased significantly, and MIS decreased versus the baseline in the IDPN group. After 6 months, serum albumin levels remained significantly higher than at baseline.^75^

11.2 **All patients on hemodialysis are recommended to have micronutrient intake according to the recommended dietary allowance, including those receiving IDPN. Patients receiving IDPN do not need additional micronutrient supplementation. (Majority agreement, 64% agreement)**

#### Rationale

All patients on hemodialysis may receive daily supplementation of water-soluble vitamins.^8^ These patients are at risk of dialysis-related depletion of all water-soluble vitamins, as well as carnitine, copper, iron, and selenium,^76^^,^^77^ In patients on hemodialysis who do not consume sufficient energy, protein, and other macronutrients, micronutrient deficiency is highly probable. For patients with an energy intake below 1500 kcal/day, especially those with a history of poor intake, oral or parenteral micronutrient supplementation may be necessary to meet the recommended dietary allowance for various micronutrients.^78^ Micronutrient deficiencies may exacerbate the effects of low serum albumin levels. Studies have shown that hypoalbuminemia and low serum zinc levels were associated with increased mortality in patients on dialysis.^79^

11.3 **IDPN may be combined with ONS or enteral nutrition to manage patients on hemodialysis with PEW. (Consensus, 82% agreement)**

#### Rationale

Although IDPN is often used in patients who cannot tolerate ONS or do not intake a sufficient amount of ONS to meet nutritional needs, several studies have successfully combined IDPN and ONS prescription for patients with PEW.^29^^,^^80^ One prospective, controlled study reported higher prealbumin values with IDPN plus ONS than with ONS alone in patients on hemodialysis.^80^ The salient feature of patients on dialysis with PEW is decreased appetite and intake, so patients fail to meet the recommended dietary intake level. At the same time, these patients are characterized by hypercatabolism, which demands increased nutritional intake.^81^ As each patient may have different nutritional needs according to the underlying causes and severity of PEW and their nutritional status, an individualized approach is needed to manage patients with PEW.

11.4 **Do not initiate IDPN in patients with volume overload, uncontrolled diabetes or hypertension, or severe hypertriglyceridemia (e.g., > 500 mg/dl [5.6 mmol/L]) until these conditions are optimized. (Consensus, 82% agreement)**

#### Practice Point

• The volume of nutritional supplementation, irrespective of type, should be ultrafiltered during dialysis sessions to maintain patient’s clinically euvolemic state and prevent volume overload. (Consensus, 82% agreement). Suggested strategies to tackle risk factors can be accessed in Supplementary Table S4.

#### Rationale

Although there are no absolute contraindications for IDPN, randomized clinical trials have been conducted using various exclusion criteria that were based on clinical experience.^2^^,^^66^ Additional intravenous volume load from IDPN may lead to uncontrolled high blood pressure and volume overload. It is important to adjust the ultrafiltration rate during hemodialysis to remove the excess volume associated with IDPN administration to minimize the risk of volume overload.^8^ Severe, uncontrolled hyperglycemia or hypertriglyceridemia, may be exacerbated by IDPN as a result of the high glucose or lipid content. However, IDPN does not seem to induce a proatherogenic lipid profile.^82^ There are no comparative data on the risk of lipid disturbances with different IDPN solutions. Based on pathophysiological considerations, lipid-free solutions,^83^ fish oil-based solutions,^72^ and dose reduction^83^ may all reduce the risk of hypertriglyceridemia. In severe hypertriglyceridemia, a lipid-free formulation may be considered. Insulin resistance is a common feature in patients with CKD and kidney failure.^84^ IDPN with high glucose content may increase the risk of hyperglycemia in patients with or without diabetes mellitus, and is generally manageable with insulin administration.^75^

12. **Administration of IDPN**
12.1 **Administer IDPN through the venous chamber of the extracorporeal circuit using an infusion pump. Avoid infusing IDPN through the arterial chamber to minimize nutrient losses. (Strong consensus, 100% agreement)**

#### Rationale

Hemodialysis eliminates small molecules and, to a lesser extent, some middle molecules. This process removes not only uremic toxins, but also amino acids and glucose. Therefore, administering parenteral nutrition before the blood passes through the dialysis membrane may result in the loss of many nutrients.^85^^,^^86^

13. **Monitoring of IDPN**
13.1 **Regularly monitor patient’s clinical status (including volume status, blood pressure, glucose levels, electrolytes, liver function, triglycerides, and acid-base status), tolerability to IDPN, and any adverse events during administration. (Strong consensus, 91% agreement)**

#### Practice Points

• It is important to look out for potential adverse effects of IDPN, hemodynamic changes, volume status, changes in various biochemical parameters including plasma glucose, lipid levels, sodium, potassium, urea, creatinine, calcium, phosphorus, magnesium, liver function, serum albumin, and acid-base status and reactive hypoglycemia.^66^ Table 4 presents the various parameters that require regular monitoring in patients receiving IDPN and the suggested frequencies of monitoring.

**Table 4:** Overview of monitoring parameters and frequency of monitoring for patients receiving IDPN^5^

Parameters	Frequency
Adverse reactions to IDPN: • Nausea, vomiting, malaise • Hypotension • Respiratory distress and/or cardiac arrhythmias (rare)	At each dialysis session Be especially vigilant during the first week of IDPN
Hemodynamic parameters: • Blood pressure • Heart rate • Volume status	At each dialysis session
Glucose metabolism: • Hyperglycemia • Hypoglycemia	• Patients with diabetes: Monitor blood glucose before dialysis, mid-session of dialysis, and at the end of dialysis • Patients without diabetes: Same monitoring schedule as for those with diabetes for the first 3 sessions unless abnormal results are observed
Biochemistry: • Complete blood count • Electrolytes (K, Ca, P, Mg, Na) • Urea, creatinine • Liver function tests (alkaline phosphatase, alanine aminotransferase [ALT], total bilirubin) • Serum albumin • Blood lipids (triglycerides and cholesterol) • Acid-base status	• Weekly before dialysis during the first 2 weeks • Thereafter, every 4–6 weeks, if stable

Ca, calcium; IDPN, intradialytic parenteral nutrition; K, potassium; Mg, magnesium; Na, natrium; P, phosphorus.
• Some patients may be advised to consume a snack rich in carbohydrates and proteins about 30 minutes before the end of the dialysis session to reduce the risk of reactive hypoglycemia, especially if insulin has been administered.
• Subcutaneous rapid-acting insulin should be given to patients on hemodialysis with diabetes receiving IDPN, whose blood glucose levels persistently exceed 250 mg/dl (13.9 mmol/l) to optimize glycemic control.
• Patients at risk of refeeding syndrome should receive 300 mg thiamine i.v., before initiating parenteral nutritional supplementation, followed by i.v., thiamine 200 to 300 mg for at least 3 more dialysis sessions.^76^ Thiamine may be supplemented daily for 7 to 10 days, with the dosage determined by clinical judgement.^87^
• A practical way to identify patients at risk of refeeding syndrome is to take a thorough medical and dietary history, especially the patient’s nutritional intake in the weeks before initiation of IDPN. The Australasian Society of Parenteral and Enteral Nutrition considers a 30% decrease in serum phosphate levels within the first 72 hours of meeting at least 50% of estimated nutrition requirements, together with other signs of phosphate imbalance, indicative of refeeding syndrome.^87^

To prevent refeeding syndrome, a very low infusion rate or a stepped prescription approach should be adopted in patients at risk, including patients with PEW, frailty, not eating for quite some duration, on dialysis, and those with poor reserves.

#### Rationale

Parenteral nutrition may alter plasma electrolytes, acid-base status, lipids, and glucose levels. Therefore, patients who receive carbohydrate-containing formulations of IDPN must be closely monitored to review if any adverse effects occur and to assess their tolerance of IDPN. Hyperglycemia and hypertriglyceridemia are potential complications in patients receiving carbohydrate-containing formulations of parenteral nutrition and are associated with poor clinical outcomes.^88^ A slow initiation of IDPN with gradual step-up of the administrated volume and frequent monitoring of plasma glucose during IDPN administration, may help prevent erratic plasma glucose levels.^89^

There is expert consensus that glycemic control in patients receiving IDPN may vary according to the glucose concentration of the IDPN formulation, the rate of IDPN administration, the patient’s fasting state, and whether the patient has underlying diabetes and has received antidiabetic drugs before the hemodialysis session. A practical approach to glycemic management is to use subcutaneous insulin during dialysis, consistent with general inpatient diabetes management.^90^ There is also a risk of rebound hypoglycemia or hyperglycemia at the end of the hemodialysis session in patients receiving IDPN, depending on the patient’s condition.

Providing nutritional support after a period of starvation may result in refeeding syndrome,^87^ particularly in vulnerable patients, for example in patients with PEW, frailty, on dialysis, and those with poor reserves. Electrolyte shifts, which are a characteristic feature of refeeding syndrome, may be exacerbated by hemodialysis in patients with poor reserves who have not eaten for a long time. For instance, hypophosphatemia in patients receiving hemodialysis is associated with poor nutritional status and muscle weakness.^91^ Although refeeding syndrome is not common with IDPN administration (as patients should have spontaneous dietary intake), it has been reported in a few patients with severe PEW and very low nutrient intake.

14. **IDPN in Patients With Hypertriglyceridemia**
14.1 **Patients with moderate to severe hypertriglyceridemia (e.g., > 500 mg/dl or 5.6 mmol/l) should receive a lipid-free IDPN formulation to prevent worsening of hypertriglyceridemia. (Consensus, 82% agreement)**

#### Rationale

Hypertriglyceridemia in patients undergoing hemodialysis is associated with an increased risk of cardiovascular mortality, including sudden cardiac death.^92^ However, hypertriglyceridemia associated with parenterally administered lipid emulsions has also been reported with the use of parenteral nutrition. In patients receiving PN, withdrawing soybean oil–based lipid emulsions for 1 to 23 days (median: 5 d) was associated with a significant reduction in plasma triglyceride levels.^83^ Therefore, temporarily withholding lipid provision might help improve hypertriglyceridemia associated with the administration of PN that contains lipid emulsions. The type of lipid emulsion may also affect triglyceride levels, as some evidence suggests that emulsions containing alternative lipid sources may result in smaller increases in triglycerides compared with soybean oil–based emulsions.^91^, ^92^, ^93^, ^94^, ^95^, ^96^ Whether these observations for TPN may also apply to IDPN remains uncertain.

## Conclusion

These consensus statements documented with practice points on ONS and IDPN, provide concise guidance for prescribing medical nutrition therapy in adult patients receiving dialysis to facilitate practical implementation of nutritional supplementation. Figure 1 presents a decision algorithm for the management of PEW and the consideration of nutritional supplementation according to patient’s clinical needs.

It is essential that patients who receive nutritional supplementation, either orally or via the parenteral route, undergo regular monitoring of their clinical and nutritional status, volume and hemodynamic status, biochemical parameters, tolerability, and potential adverse effects. A multidisciplinary approach involving nephrologists, renal dietitians, dietitians, and renal nurses, who have received appropriate training in renal nutrition, is needed to integrate and deliver medical nutrition therapy as part of the standard care in dialysis centers. It is mandatory that all health care professionals should receive appropriate training before prescribing ONS, IDPN, or other forms of medical nutrition therapy to patients receiving dialysis.

## Disclosure

AY-MW has received speaker honoraria from Astra Zeneca, Fresenius Kabi, Bayer Corporation, Boehringer Ingelheim, and Vitaflo. She served on the advisory board of Bayer Corporation, Boehringer Ingelheim, and Fresenius Kabi; is an Executive Committee member of POSIBIL6, a phase III trial sponsored by CSL Vifor; and is a scientific advisor to Consun Pharma. DF has received lecture fees and congress travel support from AstraZeneca, Boehringer, Dr Schär, Fresenius Kabi, Lilly, Astellas, and Theradial. MK has received honoraria or consulting fees from Bayer, Boehringer Ingelheim, Fresenius Kabi, and Medice. KL has received honoraria from Fresenius Kabi. PVMA has received an honorarium from Fresenius Kabi. KP has received honoraria and consulting fees from Fresenius Kabi. AS has received honoraria or consulting fees from Fresenius Kabi. YSS has received honoraria or travel support from AstraZeneca, Boehringer Ingelheim, DKSH, Fresenius Kabi, Eli Lilly, Hovid Pharma, Malaysian Society of Nephrology, Novartis, Novo Nordisk, and Servier. PM has received consulting or speaker fees from Abbott, Baxter, CSL Vifor, Fresenius Kabi, Lilly, Palex, and Toray. All authors received honoraria and travel support from Fresenius Kabi for participation as writing group members in the workshop that developed this consensus paper.

## Supplementary Material

Supplementary File (PDF) **Supplementary References. Table S1.** Summary of statements and practice points for nutritional supplementation in patients receiving dialysis. Table S2. Overview of reimbursement for ONS in selected countries worldwide. Table S3. Proposed criteria to decide on the need for initiating IDPN. Table S4. Suggested strategies to prevent or minimize potential adverse effects of IDPN.
