# Supplementary material for: An International Expert Consensus Statement and Guidance Document on Implementation of Nutritional Supplementation in Patients on Dialysis
Source: Kidney Int Rep. 2026 Jul 29;11(10):106990. doi: 10.1016/j.ekir.2026.106990 (PMC13524878; doi:10.1016/j.ekir.2026.106990)
Supplement: Supplementary file 1 [file mmc1.pdf]

# Supplemental Material

## Supplemental references

Table S1: Summary of statements and practice points for nutritional supplementation in patients receiving dialysis.

| **ONS for patients on Dialysis** | |
|---|---|
| **1. Nutrition screening for patients on dialysis** | |
| 1.1 | All patients receiving dialysis should undergo regular nutritional screening at least once every 3 months. Those at risk of PEW should undergo more frequent nutritional screening. (Strong consensus, 100% agreement) |
| 1.1.1 | Nutritional screening may be performed by any health professional who has undergone training. |
| 1.1.2 | There is no evidence of superiority of any specific nutritional screening tool in patients receiving dialysis. Commonly used tools include Geriatric Nutritional Risk Index (GNRI), Malnutrition Universal Screening Tool (MUST), Malnutrition Screening Tool (MST) and the Renal Inpatient Nutrition Screening Tool (renal iNUT). The choice of nutrition screening tools depends on local expertise and capabilities. |
| 1.2 | Those at risk of PEW or found positive with nutritional risk on screening should be referred to a renal dietitian or a dietitian trained in renal nutrition for a detailed nutritional assessment. (Strong consensus, 100% agreement) |
| 1.2.1 | Several nutrition tools may be used for nutrition assessment. The International Society of Renal Nutrition and Metabolism (ISRNM) criteria for PEW, the Malnutrition Inflammation Score (MIS), and the |

Subjective Global Assessment (SGA) are the most commonly used tools for nutritional assessments. The Global Leadership Initiative on Malnutrition (GLIM) criteria are more frequently applied in other populations such as geriatric patients. Independently of the tools used to do nutritional screening and nutritional assessment, health professionals should receive training before they apply the tools.

1.2.2 To adopt the diagnostic criteria of ISRNM, PEW is diagnosed in patients with at least one criterion in three of the four categories and at least one biochemical parameter be included in the diagnosis of PEW (Table 1).

1.2.3 The use of MIS or SGA, as presented in Figure 1, allows stratification of the severity of PEW, and helps guide the intensity and type of nutritional intervention to be adopted according to severity. In this context, mild PEW may often be managed with nutritional counselling and ONS, whereas moderate to severe PEW may require escalation towards EN, IDPN or total PN depending on the clinical scenario, spontaneous oral intake and the severity of hypercatabolic conditions

1.2.4 Nutritional assessment should be performed by registered dietitians, renal dietitians, or their international equivalents, as suggested by international guidelines.

**2. Nutritional Counseling**

2.1. Patients at risk of or confirmed with PEW on nutritional assessment should receive nutritional counselling. (Strong consensus, 100% agreement)

**3. Initiation of ONS**

3.1. Consider initiating ONS early in patients with confirmed PEW who are unable to improve or increase their spontaneous dietary intake to

meet nutritional requirements after nutritional counseling. The ONS should supplement nutritional needs of the patients, which include energy, macronutrients and micronutrients. (Strong consensus, 100% agreement)

3.1.1 Once a patient is diagnosed with PEW with reduced spontaneous oral intake that is insufficient to meet nutritional needs, ONS should be initiated.

3.1.2. Patients who are prescribed ONS should be monitored for their tolerance to ONS, clinical progress and nutritional status over time in order to tailor the formulation and prescribed amount accordingly.

3.1.3 Generally, intake of ONS may provide around 250-300 kcal energy per day.

3.1.4 It may be a challenge to administer more than 400 kcal/day orally.

3.1.5 Not all ONS are nutritionally complete, so it is crucial to select an appropriate product based on patient's specific needs, clinical conditions and any complications that may increase nutritional needs.

3.1.6 Concentrated ONS with reduced volume relative to energy and protein content may incur less volume load to patients on dialysis and reduce risk of volume overload.

3.1.7 Offering ONS products with similar nutrients content and volume but in a variety of flavors, consistencies and formats, such as low-potassium or low-phosphorus drinks, energy or protein bars, and protein powders that can be mixed into puddings, juices, or shakes, may help to reduce monotony and prevent taste fatigue, thereby enhancing adherence to ONS.

3.1.8 Home-made ONS may be considered in case commercial ONS is not available. It is important to provide close nutrition monitoring and

education on best practices to safeguard adequate nutrient content and food safety.

3.1.9 Consider patient's preferences, gut tolerance, affordability, volume status, biochemical parameters and comorbidities such as diabetes mellitus, heart failure, or volume overload when prescribing ONS to patients. The timing of ONS administration, intradialytic or interdialytic at home, should take into account patient's preference and the amount of energy and protein needed to meet the patient's nutritional needs.

**4. Renal-specific ONS**

4.1. Consider the use of high-protein, renal-specific ONS for dialysis patients with PEW, as they provide more amino acids that are needed to maintain muscle mass and replenish dialysis-related losses but with reduced volume (Strong consensus, 91% agreement)

4.1.1 Patients on dialysis who are at risk for electrolyte imbalances or volume overload may benefit from a concentrated formulation of ONS that is low in specific electrolytes and reduced in volume.

4.1.2 ONS enriched with omega-3 polyunsaturated fatty acids (PUFAs) which may facilitate achieving intakes of 1.3 to 4 g/day of long-chain omega-3 PUFAs, could potentially support patients in meeting guideline recommendations to support blood lipid management. (Majority agreement, 73% agreement)

**5. Timing of ONS**

5.1. ONS is preferably given after meals, during dialysis sessions and/or late in the evening to avoid early satiety and to maximize nutrient absorption. (Strong consensus, 100% agreement)

5.1.1 It is preferable to administer ONS one hour after meals.

5.1.2 ONS may be considered as meal replacement to prevent PEW only in very selected patients who are unable to meet their nutritional needs through conventional meals, including but not limited to those with swallowing difficulties. (Majority agreement, 73% agreement)

**6. Monitoring of Tolerance**

6.1. Tolerance and compliance to ONS should be closely monitored at initiation of ONS. (Strong consensus, 100% agreement)

6.1.1 Patients should be monitored and reassured as needed, if temporary side effects occur. They should be able to receive advice regarding issues such as difficulty tolerating the taste or texture of ONS.

6.2. In patients with poor gut tolerance or compliance issues with ONS, adjustment of the ONS formula, composition, and volume may be considered. (Strong consensus, 100% agreement)

**7. Fiber Provision**

7.1. Patients with PEW should receive dietary fiber either through diet, ONS or additional fiber supplement, in accordance with guideline recommendations to promote overall gut health. (Strong consensus, 100% agreement)

7.1.1 The amount of fiber in the diet should be checked and supplemented if necessary. When initiating fiber-containing ONS, there is an increased likelihood of flatulence or gastrointestinal discomfort. Therefore, supplementation should begin gradually, starting with one serving per day.

7.1.2 Validated methods to estimate patient's fiber intake by dietitians include 24-hour dietary recall and a 3-day or 7-day food recall or diary.

7.1.3 Total fiber intake should target the same amount as that recommended for the general population (25 to 30 g of fiber per day, depending on recommendation).

**8. Enteral Nutrition**

8.1. Consider initiation of enteral nutrition in dialysis patients who are severely malnourished and unable to reach > 20 kcal/kg/day energy intake, or > 50% of estimated energy needs through spontaneous intake plus ONS. (Strong consensus, 91% agreement)

**9. Monitoring of Nutritional Support**

9.1. Nutritional status should be monitored at least monthly after initiation of any form of nutritional support (ONS, enteral nutrition[EN], parenteral nutrition [PN], intra-dialytic parenteral nutrition [IDPN] or total parenteral nutrition [TPN]). (Strong consensus, 100% agreement)

9.1.1 After starting nutritional support, at least monthly monitoring is required. Nutritional monitoring can be integrated with tolerance checks. In patients receiving home dialysis (including PD and home HD), nutritional monitoring every 2–3 months may also be reasonable.

**10. Discontinuation of nutritional support**

10.1. Duration of ONS should be tailored according to causes of PEW, clinical targets and actual improvement. ONS are generally prescribed for a 3 to 6-month period as required. (Strong consensus, 91% agreement)

10.2. Nutritional support (either ONS, EN, PN, IDPN or TPN) may be discontinued when patients show an overall improvement in appetite, spontaneous dietary intake, weight gain, and improvement or

resolution of the clinical condition causing PEW. (Strong consensus, 100% agreement)

10.2.1 The efficacy of ONS may be monitored using the following parameters outlined in Table 2. Assessment of body composition is not mandatory but may assist in follow up of nutritional status and changes in body composition after nutritional supplementation.

## IDPN for patients on hemodialysis

### 11. Initiation of IDPN

11.1 Initiate IDPN for patients with confirmed PEW whose spontaneous dietary intake is over half of the nutritional requirements but are unable to meet total energy and protein requirement with ONS or enteral nutrition. (Strong consensus, 100% agreement)

11.1.1 Detailed criteria for the initiation of IDPN are listed in supplementary table S3. Commercially available formulations of IDPN containing all macronutrients (carbohydrate, fat and protein) are preferred over individualized parenteral nutrition formulations prepared by the hospital pharmacy because the commercially available formulations generally would meet nutritional needs of most patients on hemodialysis. The standard formulations are immediately available and do not need to be specifically prepared. They incur less preparation time and longer shelf life and lower risk of contamination. Because commercial formulations are adapted to patients’ electrolyte-free requirements and can provide high concentrations of nutrients, they help reduce the risk of electrolyte disturbances and the need for excessive ultrafiltration rates.

11.1.2 Practical administration regimen suggested: Initiate IDPN (triple phase solution that contains fat, carbohydrate and protein) at half the target volume during the first week and then gradually increase to full volume from week 2 to 4 according to patient's clinical condition, nutritional status, volume status, glycemic control, electrolyte levels and overall tolerability. (Strong consensus, 91% agreement).

Table 3 shows the prescription volume, maximal infusion rates and gradual step-up approach proposed by experts.

11.1.3 The inclusion of fish oil, a source of omega-3 polyunsaturated fatty acids (PUFAs), in the IDPN formulation may be considered, as it could be associated with potential anti-inflammatory benefits and a modest effect on triglyceride levels, although current evidence remains limited. (Consensus, 82% agreement)

11.2 All patients on hemodialysis are recommended to have micronutrients intake according to the RDA, including those receiving IDPN. Patients receiving IDPN do not need additional micronutrient supplementation. (Majority agreement, 64% agreement)

11.3 IDPN may be combined with ONS or enteral nutrition to manage patients on hemodialysis with PEW. (Consensus, 82% agreement)

11.4 Do not initiate IDPN in patients with volume overload, uncontrolled diabetes or hypertension, or severe hypertriglyceridemia (e.g., >500 mg/dL [5.6 mmol/L]) until these conditions are optimized. (Consensus, 82% agreement)

11.4.1 The volume of nutritional supplementation, irrespective of types, should be ultrafiltered during dialysis sessions, in order to maintain patients clinically euvolemic and prevent volume overload.

(Consensus, 82% agreement). Suggested strategies to tackle risk factors can be accessed in supplementary table S4.

**12. Administration of IDPN**

12.1 Administer IDPN through the venous chamber of the extracorporeal circuit using an infusion pump. Avoid infusing IDPN through the arterial chamber to minimize nutrient losses. (Strong consensus, 100% agreement)

**13. Monitoring of IDPN**

13.1 Regularly monitor patient's clinical status (including volume status, blood pressure, glucose level, electrolytes, liver function, triglyceride and acid base status), tolerability to IDPN and any adverse events during administration. (Strong consensus, 91% agreement)

13.1.1 It is important to look out for potential adverse effects with IDPN, hemodynamic changes, volume status, changes in various biochemical parameters including plasma glucose, lipids level, sodium, potassium, urea, creatinine, calcium, phosphorus, magnesium, liver function, serum albumin and acid-base status and reactive hypoglycemia. Table 4 presents the various parameters that require regular monitoring in patients receiving IDPN and suggested frequencies of monitoring.

13.1.2 Some patients may be advised to consume a snack rich in carbohydrates and proteins about 30 minutes before the end of the dialysis session to reduce the risk of reactive hypoglycemia, especially if insulin has been administered.

13.1.3 Subcutaneous rapid-acting insulin should be given in hemodialysis patients with diabetes receiving IDPN and in whom the blood glucose levels persistently exceed 250 mg/dL (13.9 mmol/L) to optimize glycemic control.

13.1.4 Patients at risk of refeeding syndrome should receive 300 mg thiamine intravenously (IV) before initiating parenteral nutritional supplementation, followed by IV thiamine 200-300 mg for at least 3 more dialysis sessions.[72] It may be supplemented daily for 7 - 10 days with the dosage determined by clinical judgement.

13.1.5 A practical way to identify patients at risk of refeeding syndrome is to take a thorough medical history and diet history especially patients' nutritional intake in the weeks before initiation of IDPN. The Australasian Society of Parenteral and Enteral Nutrition considers a 30% decrease in serum phosphate levels within the first 72 hrs. of meeting at least 50% of estimated nutrition requirements together with other signs of phosphate imbalances indicative of refeeding syndrome.

To prevent refeeding syndrome, a very low infusion rate or a stepped prescription approach should be adopted in patients at risk including patients with PEW, patients not eating for quite some duration, patients with frailty, patients on dialysis and those with poor reserves.

## 14. IDPN in Patients with Hypertriglyceridemia

14.1. Patients with moderate to severe hypertriglyceridemia (e.g. >500 mg/dL or 5.6 mmol/L) should receive lipid-free IDPN formulation to prevent worsening of hypertriglyceridemia. (Consensus, 82% agreement)

Table S2: Global overview of reimbursement and patient out-of-pocket costs related to ONS

| **A. Reimbursement of ONS in the experts' countries** | **Inpatients on maintenance dialysis** | **Outpatients on dialysis** |
|---|---|---|
| **ONS generally reimbursed** | Australia, Germany, Spain | Germany, Spain |
| **Reimbursement depends on insurance or type of hospital** | Malaysia, Mexico, USA | Australia, Malaysia, Mexico, |
| **Not reimbursed** | Philippines | Philippines, USA |

Reimbursement of ONS in the experts' countries of origin

| **B. Patient out-of-pocket expenses (USD) per dietitian/renal dietitian consultation** | **Inpatient cost Median (IQR)** | **Outpatient cost Median (IQR)** |
|---|---|---|
| **Overall** | 20 (10–30) | 20 (10–30) |
| Africa | 20 (13–25) | 25 (20–40) |
| Eastern and Central Europe | 30 (25–35) | 33 (30–35) |
| Latin America and the Caribbean | 40 (1–40) | 18 (6–38) |
| Middle East | 30 (30–30) | 30 (5–50) |
| North and East Asia | 10 (10–15) | 10 (10–10) |
| Oceania and Southeast Asia | 15 (10–20) | 16 (9–20) |
| South Asia | 10 (3–20) | 10 (5–10) |
| Western Europe | 8 (8–8) | 17 (8–25) |
| | | |
| Low-income countries | 30 (30–30) | 18 (5–30) |
| Lower-middle-income countries | 10 (5–20) | 11 (8–18) |
| Upper-middle-income countries | 30 (10–35) | 20 (5–35) |
| High-income countries | 20 (10–30) | 20 (10–30) |

Global comparison of patient out-of-pocket expenses (USD) per dietitian/renal dietitian consultation.[1] Data is based on a survey by the International Society of Nephrology (ISN) among nephrology leaders in 182 countries. Abbreviations: USD, US dollar; IQR, interquartile range

Table S3: Proposed criteria to decide on the need for initiating IDPN.[2]

| **Criteria for the initiation of IDPN** | |
|---|---|
| Patients on hemodialysis who meet all of the following criteria: | • Spontaneous intake over 50% of nutritional targets<br>• Failure to improve spontaneous intake despite intensive nutritional counseling and ONS<br>• Patients with or at risk of PEW |
| Patients on hemodialysis who, despite receiving oral supplementation, meet 3 or more of the following additional criteria: | • Mean albumin ≤3.8 g/dL or prealbumin <20 mg/dL levels, for the last three months<br>• Serum creatinine <8 mg/dL, for the last three months<br>• Unintentional weight loss >10% and 20% of their usual and ideal weight, respectively<br>• BMI <18.5 kg/m²<br>• Loss of lean tissue mass (documented by bioimpedance spectroscopy or ultrasound)<br>• SGA score B or C (moderate-severe malnutrition)<br>• Decreased intake without achieving caloric needs (<25–30 kcal/kg/day)<br>• Decreased intake without meeting protein needs (<1.0 g/kg/day) |

Abbreviations: BMI = Body mass index, ONS = oral nutritional supplements, PEW = protein energy wasting, SGA = subjective global index

Table S4: Suggested strategies to prevent or minimize potential adverse effects of IDPN

| **Potential adverse effect** | **Proposed handling** |
|---|---|
| Volume overload | • Ensure the volume of IDPN is included in UF volume<br>• UF based on dry weight estimate after dialysis<br>• Use diuretics to optimize urine volume in patients with residual kidney function<br>• Use dialysate with sodium content close to plasma sodium levels of the patient<br>• Minimize volume of other infusions<br>• Consider concentrated ONS (defined as ≥2kcal energy/ml) |
| Uncontrolled diabetes | • Consider subcutaneous rapid-acting insulin,<br>• Consider IDPN with low glucose content or lipid only solutions |
| Hypertension | • Adapt ultrafiltration rate to achieve euvolemia<br>• Ensure adherence to reduced sodium diet (<100mml/day) and fluid restricted diet<br>• Schedule antihypertensive drugs after dialysis<br>• Consider IDPN with low sodium and glucose content |
| Hypertriglyceridemia | • Consider reduced IDPN dose (carbohydrate load)<br>• Use lipid-free formulation of IDPN<br>• Consider IDPN with reduced glucose content and IDPN with omega-3 fatty acids |

| | |
|---|---|
| | • Consider statins or fibrates |

Abbreviations: IDPN = intradialytic parenteral nutrition, UF = ultrafiltration

# 1 References

S1 Wang AYM, Okpechi IG, Ye F et al. Assessing Global Kidney Nutrition Care. *Clin J Am Soc Nephrol 2022;*17(1):38–52. DOI: 10.2215/CJN.07800621.

S2 Molina P, Quilis A, Durbá A, et al. A roadmap for implementing a successful clinical experience with intradialytic parenteral nutrition. *Clin Nutr ESPEN*. 2024;63:322-331. doi:10.1016/j.clnesp.2024.06.044
